# Anesthetic drug concentrations and placental transfer rate in fetus between term and preterm infants, twins, and singletons

**DOI:** 10.3389/fphar.2023.1213734

**Published:** 2023-09-01

**Authors:** Hao Liu, Jing-Kun Miao, Meng Cai, Lin Gan, Hui-Qing Zhao, Xiao-Feng Lei, Jin Yu

**Affiliations:** ^1^ Department of Pediatrics, Chongqing Health Center for Women and Children, Women and Children’s Hospital of Chongqing Medical University, Chongqing, China; ^2^ Department of Anesthesiology, Chongqing Health Center for Women and Children, Women and Children’s Hospital of Chongqing Medical University, Chongqing, China

**Keywords:** cesarean section, general anesthesia, preterm, twins, drug concentration

## Abstract

**Objective:** This study aims to determine the drug concentration of etomidate, remifentanil, and rocuronium bromide for general anesthesia in fetus as well as the placental transport rate between term and preterm delivery, twins, and singleton.

**Study design:** Sixty parturients with 72 fetuses undergoing cesarean section under general anesthesia were included. According to whether the fetus was a twin or premature, parturients were divided into Group I (term singleton), Group II (premature singleton), Group III (term twins), and Group IV (premature twins). The preoperative demographic characteristics and laboratory examination of parturients, hemodynamic indicators, the Apgar score of neonates at 1, 5, and 10 min after delivery and at specific assigned values, umbilical artery blood gas analysis results, neonatal weight, and resuscitative measures were recorded. Anesthetic drug concentrations in maternal arterial (MA), umbilical arterial (UA), and umbilical venous (UV) blood were detected by Ultra Performance Liquid Chromatography Tandem Mass Spectrometry (UPLC-MS/MS).

**Result:** No significant differences were observed in the concentrations of etomidate, remifentanil, and rocuronium bromide in MA, UV, and UA blood, or in the UV/MA and UA/UV ratios between term and preterm infants, twins, and singletons. Moreover, there was no variation in the anesthetic drug concentration among each pair of twins. Additionally, no correlation was found between the neonatal weight and the plasma concentrations of anesthetic drugs in UV and UA blood, except for remifentanil in UA blood.

**Conclusion:** Preterm or twin deliveries do not affect the neonatal concentration of etomidate, remifentanil, and rocuronium bromide used in general anesthesia for cesarean sections.

**Clinical Trial Registration:** www.chictr.org.cn, identifier ChiCTR2100046547

## 1 Background

Intraspinal anesthesia remains the preferred option for cesarean sections, surpassing general anesthesia in numerous aspects. Nonetheless, in certain critical cases, general anesthesia may be more suitable or had to be the sole alternative. Recent reports indicate that general anesthesia has accounted for 5.8% of total cesarean section anesthesia and 14.6% of emergency cesarean sections due to various reasons (Juang et al., 2017). In terms of general anesthesia, etomidate, remifentanil, and rocuronium bromide are widely used in practice.

Remifentanil belongs to μ opioid receptor agonists and is administered intravenously during anesthesia for cesarean section. It offers an optimal balance between reducing maternal stress response and minimizing neonatal respiratory depression (White et al., 2019). Remifentanil easily passes through the placental barrier due to its fat-soluble characteristics. However, it can be hydrolyzed in cord blood due to its rapid metabolism features and will rapidly be redistributed and can even enter the fetus. The distribution volume of remifentanil in newborns and infants is larger than that in adults or toddlers, and the clearance rate is faster (Kan et al., 1998)**.** Our previous data demonstrate that there is no correlation between the plasma concentration of remifentanil in full-term newborns and induction to delivery (I-D) intervals time. This finding is consistent with another study (Hu et al., 2017) that reported that the mean concentrations of remifentanil in the MA, UA, and UV blood at delivery in the shorter I-D group were similar to those in the longer I-D group.

Etomidate has been used in cesarean sections under general anesthesia for approximately 50 years (Downing et al., 1979). In particular, etomidate offers several advantages for parturients with previous congenital heart disease (Clivatti et al., 2012; Ho et al., 2018). Remifentanil combined with etomidate and muscle relaxant (rocuronium bromide) are becoming much more popular for general anesthesia in cesarean sections; however, the concentration and placental transfer of these three drugs in twins and premature infants is poorly understood.

Due to advancements in assisted reproductive technology, the incidence of twin pregnancies has escalated significantly. It is noteworthy that twin pregnancies have a higher likelihood of premature birth compared to singleton pregnancies. Premature newborns and full-term newborns exhibit significant differences in many aspects of organ development, and the risk factors affecting respiratory depression also vary between them (Condo et al., 2017). Recent research (Louchet et al., 2021) indicates that cord concentrations of antiretroviral differed in nearly one-third of twins in the same pair, which may be due to inter-individual genetic variability of placental transporters or physiological differences between twins.

For general anesthesia drugs, concerns are arising regarding the side effects of these anesthetic agents for neonates due to the lack of well-established dosing regimens for these drugs in the specific population. It remains unclear whether there are variations in drug concentrations or placental transport rates between term and preterm newborns as well as between twins. The primary objectives of this study were to ascertain the drug concentrations in the fetus and the placental transport rates for twin pairs and preterms versus full-term deliveries during cesarean sections performed under general anesthesia. This research has potential implications in clinics for understanding how anesthesia drug administration during cesarean sections affects fetal exposure to medications, especially in the context of multiple pregnancies and premature births.

## 2 Materials and methods

### 2.1 Study subjects

**FIGURE 1:**
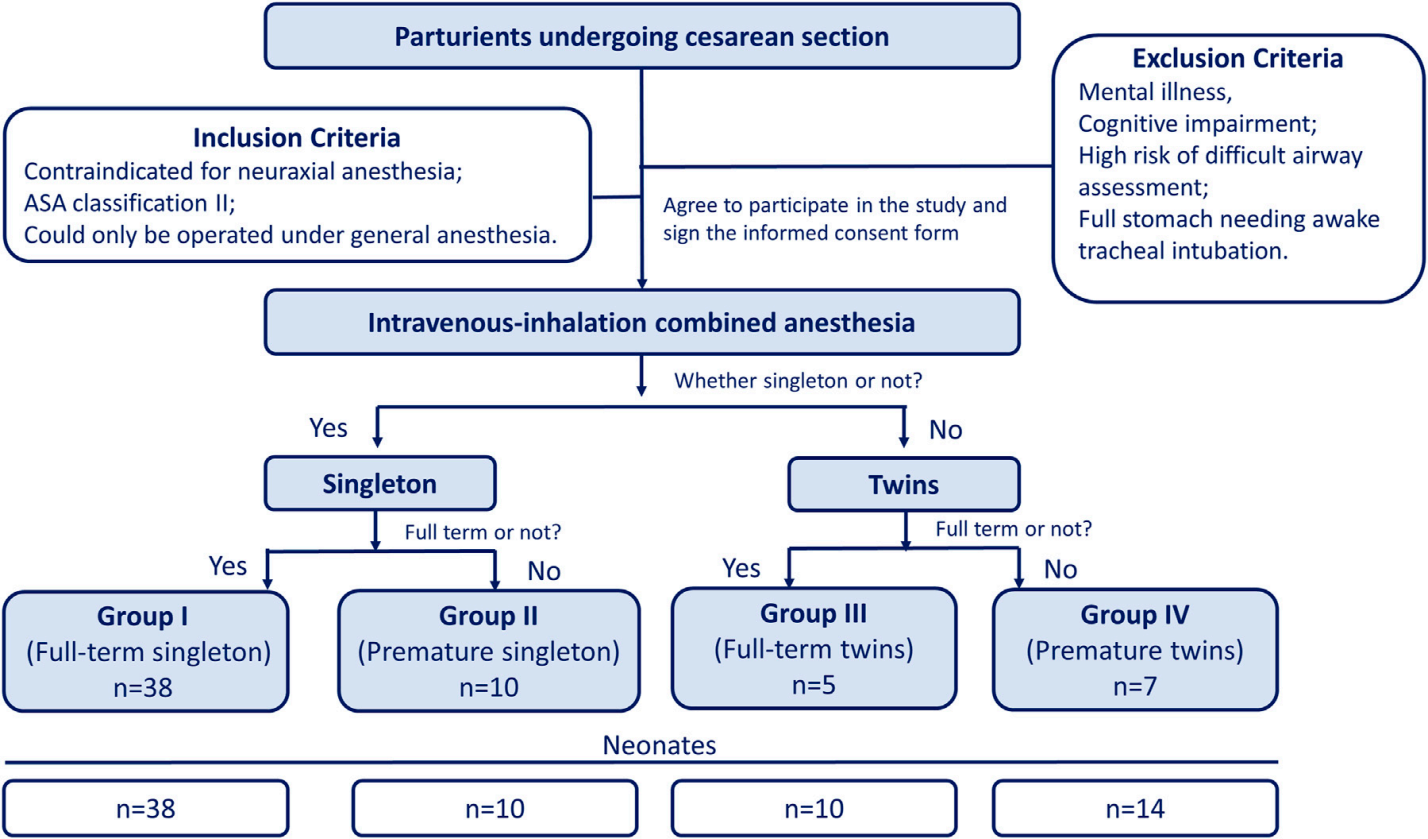
Flow diagram of this clinical study.

This study was implemented in accordance with the Declaration of Helsinki, was approved by the Research Ethics Committee of the Women and Children’s Hospital of Chongqing Medical University (Number 2021-011), and was registered with the Chinese Clinical Trial Registry (ChiCTR) (www.chictr.org) (registration ID: ChiCTR2100046547). Informed consent was obtained from all participants. The inclusion criteria in this study are parturients with a preoperative history of lumbar disc herniation or lumbar surgery, thrombocytopenia or other medical conditions that contraindicated to intraspinal anesthesia. Sixty parturients with 72 fetuses undergoing cesarean section under intravenous–inhalation combined anesthesia were included. The exclusion criteria for this study were as follows: mental illness, cognitive impairment, high risk of difficult airway assessment, or full stomach needing awake tracheal intubation. According to whether the parturient was twin or premature, enrolled parturients were divided into four groups as follows: Group I (full-term singleton), Group II (premature singleton), Group III (full-term twins), and Group IV (premature twins) (see Figure 1).

### 2.2 Anesthesia management

All parturients were strictly fasted for at least 8 h and forbidden to drink for 2 h before surgery. After entering the operating room, all parturients lay on their left side at 30° to reduce the compression of the inferior vena cava and abdominal aorta by the uterus and to prevent the occurrence of supine hypotension syndrome. Firstly, a peripheral venous route in the forearm was established. Parturients were administered oxygen via a face mask and non-invasive arterial pressure (NIBP), heart rate (HR), and peripheral oxygen saturation (SpO_2_) were monitored as normal. Anesthesia was induced with target-controlled infusion (TCI) of remifentanil at 5 ng/mL combined with 5% sevoflurane inhalation (sevoflurane was administered for a brief duration and was discontinued if the parturients lost consciousness). In the meantime, etomidate (0.25 mg/kg) and rocuronium (0.6 mg/kg) were administered intravenously. Once the muscle relaxant rocuronium took effect, endotracheal intubation was inserted and connected with an anesthesia machine for mechanical ventilation. Anesthesia was maintained using remifentanil (TCI, 5 ng/mL) and propofol (TCI, 3.5 μg/mL) after umbilical cord ligation.

### 2.3 Data collection

The recorded data for statistical comparisons include the preoperative demographic characteristics and laboratory examination of parturients, hemodynamic indicators, the Apgar score at 1, 5, and 10 min after delivery, umbilical artery blood gas analysis, neonatal weight, and resuscitative measures, including tactile stimulation and bag-mask ventilation or tracheal intubation of newborns. Maternal arterial (MA), umbilical arterial (UA), and umbilical venous (UV) blood samples were obtained after umbilical cord ligation for analysis of drug concentrations. The samples were centrifuged in sodium citrate–coated anticoagulant tubes at 3,000 rpm at 4°C for 10 min. The plasma was isolated and stored at −80°C for later analysis.

### 2.4 Chemicals and reagents

The following chemicals were used for the analysis procedure: methanol and acetonitrile were used for Ultra Performance Liquid Chromatography (UPLC) (Fisher Scientific, United States), remifentanil standard was purchased from Stanford Chemicals (FJTI-8736, Oregon, United States), etomidate (E933300) and rocuronium (R639500) standards were purchased from Toronto Research Chemicals (Toronto, Canada), internal standards (IS) sufentanil was purchased from Stanford Chemicals (SREP-5931, Oregon, United States), metomidate was purchased from MACKLIN (Shanghai, China), vecuronium (V102500) was purchased from Toronto Research Chemicals (Toronto, Canada), Milli-Q apparatuses (Millipore, Bedford, MA, United States) were used to purify the water during the study, and formic acid was purchased from Shanghai Aladdin Bio-Chem Technology Co., LTD. (Shanghai, China).

### 2.5 Analyses based on liquid chromatography-mass spectrometry Apparatus and UPLC-MS/MS

A tandem mass spectrometer (Waters, United States) equipped with an electrospray ionization source operated in the positive mode provided the analytical platform. A reverse-phase BEH C18 column (50 × 2.1 mm, 1.7 m, Waters Acquity) was used for separation. This was conducted at 45 °C with a 0.4 mL/min flow of milli-Q water containing 0.1% (v/v) formic acid (solution A) along with a 1:1 (v/v) mixture of methanol and acetonitrile containing 0.1% (v/v) formic acid (solution B). From 0 to 0.6 min, the gradient was 10% solution B; from 0.6 to 2.8 min, 10%–90% solution B; and from 2.8 to 3.6 min, 10% solution B.

### 2.6 Multiple reaction monitoring (MRM) mode of mass spectrometry

Using the tandem mass spectrometer as an electrospray ionization source, eluted drugs were analyzed at 150°C; capillary voltage, 3.0 kV; desolvation temperature, 500°C; gas flow for desolvation, 800 L/h; and gas flow for cone, 150 L/h. An MRM event was recorded every time-scheduled minute. Data were processed and quantified using MassLynx mass spectrometry software (Waters, Massachusetts, United States). The three target drugs were identified using structurally similar drugs of protonated molecules and by comparing their retention times with those of IS. As part of the MRM mode, extracted ion chromatograms were created using the mass of the precursor ions that collided in the collision chamber to produce fragment ions. The most stable fragment ion with the highest intensity was used for quantification, from which the area under the peak was calculated.

### 2.7 Statistical analysis

Statistical analyses were performed using SPSS version 22.0 for Windows. Measurement data are expressed as the mean ± standard deviation ( $\bar{x}$ ±s) and count data are expressed in actual numbers. The measurement data were compared between the groups using one-way ANOVA, and Brown-Forsythe ANOVA was used for heteroscedasticity indicators. The count data were compared using the chi-square test or Fisher’s exact test. As for *post hoc* tests, Bonferroni was used for data with homogeneous variance, and Tamhane’s was used for data with uneven variance. Correlation of the neonatal weight with the plasma concentrations of anesthetic drugs was evaluated using a Spearman rank correlation test. Values of *p* < 0.05 were considered to indicate statistical significance.

## 3 Results

### 3.1 Preoperative general information and laboratory examination of parturients

There was no statistically significant difference among the four groups in preoperative general information, except for gestational weeks. For preoperative laboratory examination of parturients, the D-dimer of Group IV was nearly twice as high as that of Group I (*p* < 0.05). These differences were related to the grouping criteria. Table 1 shows the comparison of preoperative general information and laboratory examination of parturients among the four groups. The hemodynamic indicators, including HR, NIBP, and SpO_2_, are shown in Supplementary Table S1.

**TABLE 1:** General information and laboratory examination of parturients.

Parameters	Group I (n = 38)	Group II (n = 10)	Group III (n = 5)	Group IV (n = 7)	*p*-Value
	Full-term singleton	Premature singleton	Term twins	Premature twins	
**General information**					
Age, years	32.24 ± 3.87	28.50 ± 2.99	32.00 ± 3.39	30.86 ± 5.49	0.071
Body weight, kg	72.07 ± 11.19	65.76 ± 10.08	74.40 ± 6.58	67.73 ± 8.88	0.270
Height, cm	158.11 ± 5.09	158.00 ± 5.42	157.00 ± 4.36	155.71 ± 6.75	0.724
BMI	28.77 ± 3.92	26.42 ± 4.47	30.25 ± 3.39	28.02 ± 4.42	0.289
Gestation, weeks	38.82 ± 0.86	34.60 ± 2.03^a^	37.11 ± 0.25^ab^	34.29 ± 1.89^ac^	0.000
Gestational hypertension (Yes/No)	0/38	1/9	0/5	1/6	0.141
Gestational diabetes mellitus (Yes/No)	7/31	1/9	1/4	3/4	0.396
Scar uterus (previous CS) (Yes/No)	15/23	2/8	1/4	1/6	0.393
**Laboratory examination**					
Hb^a^, g/L	125.63 ± 9.88	125.10 ± 13.15	117.40 ± 21.84	121.43 ± 14.18	0.482
Hct^a^, %	36.23 ± 4.19	36.59 ± 3.55	34.18 ± 5.29	35.19 ± 3.58	0.668
Leukocyte, 10^9^/L	8.07 ± 1.97	8.90 ± 1.30	7.38 ± 1.44	7.97 ± 2.50	0.486
PLT^a^, 10^9^/L	140.26 ± 57.92	163.90 ± 58.85	148.40 ± 66.44	150.53 ± 66.72	0.731
PT^a^, s	10.37 ± 1.36	10.20 ± 0.79	10.20 ± 0.45	10.29 ± 0.76	0.974
APTT^a^, s	26.09 ± 1.99	26.01 ± 1.30	28.60 ± 3.09	26.74 ± 0.75	0.050
FIB^a^, g/L	4.60 ± 0.72	4.55 ± 0.90	4.82 ± 0.72	4.17 ± 1.44	0.577
TT^a^, s	17.47 ± 0.76	17.20 ± 0.92	17.80 ± 0.84	19.43 ± 2.64	0.070
D-Dimer, mg/L	2.43 ± 1.29	2.46 ± 1.86	4.11 ± 2.44	4.40 ± 2.32^a^	0.010
**I-S** ^a^, (min)	2.62 ± 0.48	2.76 ± 0.68	2.29 ± 0.59	2.26 ± 0.54	0.164
**U-D** ^a^, (second)	60.21 ± 25.10	55.30 ± 36.79	66.20 ± 15.01	46.86 ± 7.58	0.532
**I-D** ^a^, (min)	6.13 ± 2.00	7.13 ± 4.74	4.95 ± 0.75	5.19 ± 1.72	0.461

^a^ Hb, Hemoglobin, Hct:Hematocrit, PLT:platelet, PT, plasma prothrombin time, APTT, activated partial thrombin time, FIB, plasma fibrinogen, TT, thrombin time, I-S, induction to skin incision time (min), U-D, uterine incision to delivery time (second), I-D, anesthesia induction to delivery intervals (min).

a: Compared with Group I, *p* < 0.05; b: Compared with Group II, *p* < 0.05; c: Compared with Group III, *p* < 0.05.

### 3.2 Neonatal-related indicators

A total of 60 parturients and 72 newborns were included in this analysis. The weight of singleton premature newborns and twin newborns was significantly lower than that of the singleton term group (*p* < 0.05). The incidence of neonatal asphyxia in Group IV (35.71%) was significantly higher than that in full-term delivery groups (I, III) (*p* < 0.05). Although there was no statistically significant difference in 1-5-10 min Apgar scores among the groups, when the sub-items of the score were compared, the reflex and color scores of the 1-min Apgar scores in Group IV were worse than those in other groups (*p* < 0.05). Additionally, the color scores of the 5-min Apgar scores in Groups II and IV were also worse than those in Group I (*p* < 0.05). The proportion of bag-mask ventilation usage for newborns and the send to NICU rate in premature newborns (Groups II and IV) were higher than those in term newborns (Groups I and III). The proportion of tracheal intubation in Group IV was significantly higher than that in Group I (*p* < 0.05). The results of the blood gas analysis were consistent with the neonatal condition; the umbilical artery blood pH of Group IV was significantly lower than that of Group I (*p* < 0.05) (see Table 2).

**TABLE 2:** Neonatal related indicators of four groups.

Parameters	Group I (n = 38)	Group II (n = 10)	Group III (n = 10)	Group IV (n = 14)	*p*-Value
	Full-term singleton	Premature singleton	Term twins	Premature twins	
Weight, g	3397 ± 452	2359 ± 536^a^	2544 ± 260^a^	2102 ± 357 ^ac^	0.000
Neonatal asphyxia	2 (5.25%)	3 (30%)	0 (0%)	5 (35.71%) ^ac^	0.008
**1 min Apgar scores**	9.53 ± 1.03	8.40 ± 1.84	9.80 ± 0.42	8.07 ± 2.62	0.007
Muscle tone (0/1/2)	0/5/33	0/4/6	0/1/9	1/4/9	0.117
Heart rate (0/1/2)	0/1/37	0/1/9	0/0/10	0/2/12	0.276
Reflex (0/1/2)	0/0/38	0/1/9	0/0/10	0/3/11^a^	0.016
Color (0/1/2)	0/5/33	0/4/6	0/1/9	0/7/7^a^	0.014
Respiration (0/1/2)	1/5/32	1/4/5	0/0/10	2/3/9	0.056
**5 min Apgar scores**	9.92 ± 0.36	9.50 ± 1.08	10.00 ± 0.00	9.27 ± 1.49	0.049
Muscle tone (0/1/2)	0/2/36	0/2/8	0/0/10	0/2/12	0.218
Heart rate (0/1/2)	0/0/38	0/0/10	0/0/10	0/0/14	—
Reflex (0/1/2)	0/0/38	0/0/10	0/0/10	0/1/13	0.472
Color (0/1/2)	0/0/38	0/2/8^a^	0/0/10	0/4/10^a^	0.002
Respiration (0/1/2)	0/1/37	0/1/9	0/0/10	1/1/12	0.328
**10 min Apgar scores**	9.97 ± 0.16	9.80 ± 0.42	10.00 ± 0.00	9.71 ± 0.83	0.163
Muscle tone (0/1/2)	0/1/37	0/2/8	0/0/10	0/1/13	0.147
Heart rate (0/1/2)	0/0/38	0/0/10	0/0/10	0/0/14	—
Reflex (0/1/2)	0/0/38	0/0/10	0/0/10	0/0/14	—
Color (0/1/2)	0/0/38	0/0/10	0/0/10	0/0/14	—
Respiration (0/1/2)	0/0/38	0/0/10	0/0/10	1/1/12	0.219
Tactile stimulation	38	10	10	14	—
Bag-mask ventilation	4	6^a^	0^b^	6 ^ac^	0.001
Tracheal intubation	0	1	0	3^a^	0.019
NICU (Yes/No)	5/33	9/1^a^	1/9^b^	12/2^ac^	0.000
UA blood pH	7.28 ± 0.05	7.26 ± 0.05	7.27 ± 0.05	7.23 ± 0.04^a^	0.037
UA blood PaO_2_ (mmHg)	31.69 ± 13.03	29.70 ± 13.37	31.38 ± 8.50	28.79 ± 12.51	0.898
UA blood PaCO_2_, (mmHg)	55.17 ± 7.55	57.79 ± 9.08	49.00 ± 7.03	56.14 ± 8.46	0.125
UA blood Base excess (mmol/L)	−2.04 ± 1.89	−2.46 ± 2.51	−4.86 ± 1.55^a^	−4.75 ± 2.96^a^	0.001

NICU, neonatal intensive care unit, UA, umbilical artery.

a: Compared with Group I, *p* < 0.05; b: Compared with Group II, *p* < 0.05; c: Compared with Group III, *p* < 0.05.

### 3.3 Plasma concentrations of general anesthetics

Twin birth and premature delivery had no effect on the concentrations of general anesthetics at delivery. There was no significant difference in the plasma concentrations of the anesthetic drugs etomidate, remifentanil, and rocuronium bromide in MA, UV, and UA blood as well as the UV/MA ratio and UA/UV ratio among the four groups (*p* > 0.05) (see Tables 3–5). Among the 12 twin parturients, only 2 of them were monochorionic diamniotic, while the remaining 10 were dichorionic diamniotic. As shown in Supplementary Table S2, there were no statistically significant differences between monochorionic diamniotic and dichorionic diamniotic at the three anesthetics concentrations in MA, UV, and UA blood.

**TABLE 3:** Blood concentration of etomidate (ng/mL).

Parameters	Group I (n = 38)	Group II (n = 10)	Group III (n = 10)	Group IV (n = 14)	*p*-Value
	Full-term singleton	Premature singleton	Term twins	Premature twins	
MA, ng/mL	432.57 ± 143.28	411.92 ± 196.94	508.16 ± 43.82	573.98 ± 221.59	0.126
UV, ng/mL	307.99 ± 12.42	290.48 ± 156.47	372.61 ± 119.60	315.08 ± 130.32	0.491
UA, ng/mL	181.28 ± 71.07	177.42 ± 123.30	201.98 ± 80.99	165.51 ± 42.12	0.725
UV/MA ratio	0.73 ± 0.19	0.69 ± 0.14	0.72 ± 0.19	0.60 ± 0.25	0.219
UA/UV ratio	0.62 ± 0.18	0.66 ± 0.24	0.57 ± 0.21	0.60 ± 0.26	0.831

MA, maternal arterial, UA, umbilical arterial, UV, umbilical venous.

**TABLE 4:** Blood concentration of remifentanil (ng/mL).

Parameters	Group I (n = 38)	Group II (n = 10)	Group III (n = 10)	Group IV (n = 14)	*p*-Value
	Full-term singleton	Premature singleton	Term twins	Premature twins	
MA, ng/mL	4.00 ± 1.53	4.34 ± 1.20	3.95 ± 0.91	3.31 ± 1.12	0.524
UV, ng/mL	2.05 ± 0.94	1.72 ± 0.78	1.61 ± 0.93	1.60 ± 0.43	0.195
UA, ng/mL	1.09 ± 0.74	0.87 ± 0.68	0.91 ± 1.01	0.50 ± 0.41	0.090
UV/MA ratio	0.51 ± 0.15	0.41 ± 0.17	0.40 ± 0.17	0.51 ± 0.21	0.111
UA/UV ratio	0.50 ± 0.24	0.48 ± 0.24	0.46 ± 0.34	0.36 ± 0.30	0.394

MA, maternal arterial, UA, umbilical arterial, UV, umbilical venous.

**TABLE 5:** Blood concentration of rocuronium bromide (μg/mL).

Parameters	Group I (n = 38)	Group II (n = 10)	Group III (n = 10)	Group IV (n = 14)	*p*-Value
	Full-term singleton	Premature singleton	Term twins	Premature twins	
MA, ug/mL	5.59 ± 1.91	6.41 ± 3.41	5.50 ± 1.21	7.12 ± 3.74	0.407
UV, ug/mL	0.84 ± 0.55	0.65 ± 0.25	0.98 ± 0.70	0.67 ± 0.43	0.387
UA, ug/mL	0.50 ± 0.34	0.42 ± 0.16	0.50 ± 0.35	0.35 ± 0.18	0.370
UV/MA ratio	0.16 ± 0.09	0.14 ± 0.09	0.17 ± 0.09	0.13 ± 0.13	0.640
UA/UV ratio	0.60 ± 0.22	0.66 ± 0.17	0.53 ± 0.10	0.58 ± 0.17	0.483

MA, maternal arterial, UA, umbilical arterial, UV, umbilical venous.

### 3.4 Comparison of plasma concentration between twins

During a cesarean section of a twin pregnancy, the birth order has no impact on the blood concentration of anesthetic drugs (etomidate, remifentanil, and rocuronium bromide) in UV or UA blood and UV/MA ratio and UA/UV ratio (*p* > 0.05) (see Table 6).

**TABLE 6:** Comparison of plasma concentration between twins.

Parameters	The first of the twins in Group III (n = 5)	The second of the twins in Group III (n = 5)	The first of the twins in Group IV (n = 7)	The second of the twins in Group IV (n = 7)	*p*-Value
**Etomidate**					
UV, ng/mL	400.23 ± 90.87	345.00 ± 148.39	346.30 ± 138.41	283.85 ± 123.95	0.939
UA, ng/mL	239.16 ± 93.26	164.80 ± 51.04	167.32 ± 46.32	163.69 ± 41.12	0.599
UV/MA ratio	0.78 ± 0.13	0.66 ± 0.23	0.67 ± 0.29	0.53 ± 0.21	0.533
UA/UV ratio	0.60 ± 0.18	0.54 ± 0.26	0.55 ± 0.28	0.65 ± 0.26	0.897
**Remifentanil**					
UV, ng/mL	1.74 ± 1.06	1.47 ± 0.88	1.60 ± 0.50	1.53 ± 0.39	0.496
UA, ng/mL	1.00 ± 1.08	0.82 ± 1.05	0.51 ± 0.41	0.48 ± 0.44	0.131
UV/MA ratio	0.42 ± 0.18	0.37 ± 0.17	0.52 ± 0.23	0.50 ± 0.21	0.338
UA/UV ratio	0.47 ± 0.29	0.45 ± 0.41	0.37 ± 0.29	0.35 ± 0.33	0.867
**Rocuronium bromide**					
UV, ng/mL	1.13 ± 0.78	0.83 ± 0.68	0.73 ± 0.56	0.60 ± 0.29	0.482
UA, ng/mL	058 ± 0.36	0.43 ± 0.36	0.35 ± 0.23	0.34 ± 0.13	0.453
UV/MA ratio	0.20 ± 0.10	0.14 ± 0.09	0.16 ± 0.18	0.10 ± 0.06	0.550
UA/UV ratio	0.54 ± 0.14	0.52 ± 0.06	0.55 ± 0.17	0.60 ± 0.18	0.774

MA, maternal arterial, UA, umbilical arterial, UV, umbilical venous.

### 3.5 Correlation between drug concentration and neonatal weight

**FIGURE 2:**
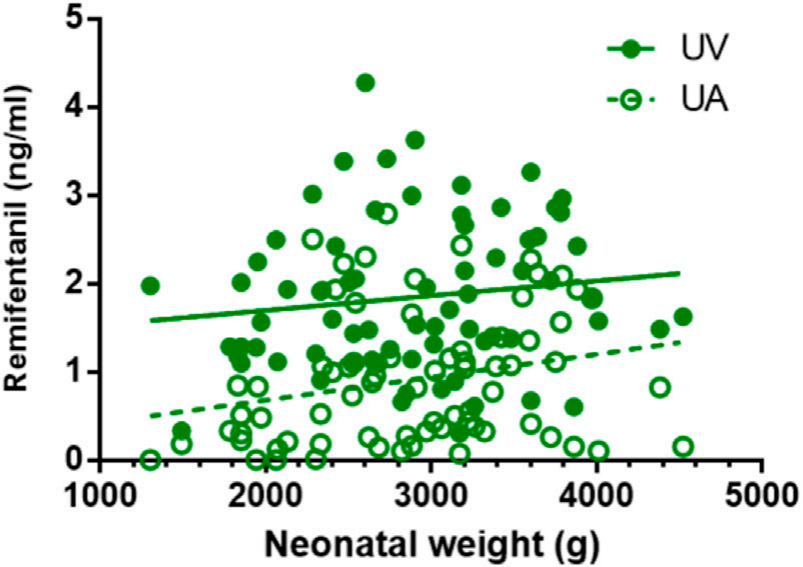
Correlations of neonatal remifentanil concentrations and neonatal weight. Ther is no correlation between neonatal weight and neonatal remifentanil plasma concentration in UV blood. There is a positive correlation between neonatal weight and remifentanil plasma concentrations in UA blood (*p* < 0.05).

**FIGURE 3:**
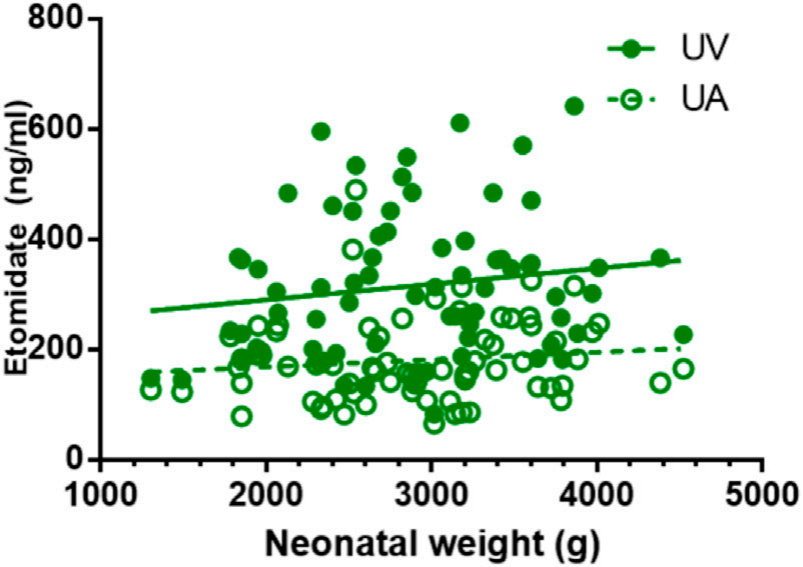
Correlations of neonatal etomidate concentrations and neonatal weight. There is no correlation between neonatal weight and etomidate plasma concentration in UV and UA blood (*p* > 0.05).

**FIGURE 4:**
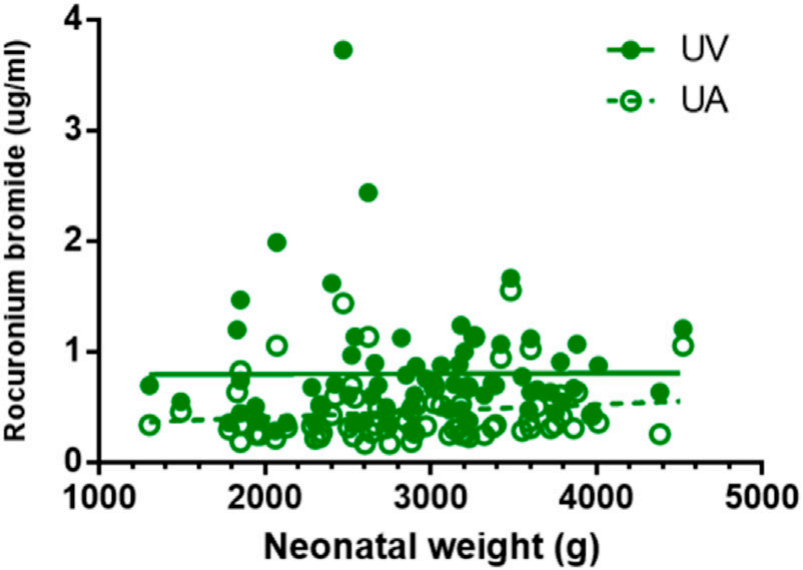
Correlations of neonatal rocuronium bromide concentrations and neonatal weight. There is no correlation between neonatal weight and rocuronium bromide plasma concentration in UV and UA blood (*p* > 0.05).

Except for remifentanil in UA blood, neonatal weight showed no significant correlation with the plasma concentration of anesthetic drugs in UV or UA blood (see Figures 2–4). A positive correlation was found between remifentanil plasma concentrations and neonatal weight in UA blood (*p* < 0.05) (see Table 7).

**TABLE 7:** Correlation coefficient of neonatal blood concentrations of etomidate, remifentanil, rocuronium bromide with neonatal weight.

Drugs	UV (r, *p*-value)	UA (r, *p*-value)
Etomidate	0.155, *p* = 0.193	0.123, *p* = 0.305
Remifentanil	0.138, *p* = 0.249	0.244, *p* = 0.039
Rocuronium bromide	0.006, *p* = 0.958	0.135, *p* = 0.256

## 4 Discussion

The utilization of opioid induction for CS remains a controversial topic, with numerous theorized risks and benefits. The rising incidence of caesarean deliveries for premature babies under general anesthesia raises concerns about the assumption that neonate exposure to anesthetics will lead to respiratory depression after birth. Cord blood concentrations reflect the drug transfer across the placenta and pharmacokinetics in the fetus, both of which may differ within a twin pair or between preterm and term infants. In this study, the blood concentrations of various anesthetic drugs (etomidate, remifentanil, and rocuronium bromide) in MA, UV, and UA blood at delivery undergoing caesarean section between term and preterm infants, twins, and singletons were analyzed by UPLC-MS/MS. No differences were found for these anesthetic drugs concentrations in MA, UV, and UA blood or UV/MA and UA/UV ratios between term and preterm infants, twins, and singletons. Moreover, there was no variation in the anesthetic drug concentration among each pair of twins. Additionally, no correlation was found between the neonatal weight and the plasma concentrations of anesthetic drugs in UV and UA blood, except for remifentanil in UA blood.

Our data reveals that the incidence of neonatal asphyxia in twin premature delivery was up to 35.71% for caesarean section under general anesthesia. Immediately after birth, we observed that the reflex and color of newborns were predominantly affected, while respiration seemed less impacted. This suggests that the adverse effects on preterm twin infants due to general anesthesia may not be directly attributed to respiratory depression caused by the anesthesia drugs. In addition, the proportion of tracheal intubation in the premature twin group was significantly higher than that in the full-term singleton group, suggesting extra attention should be paid to the issue of asphyxia in newborns born through cesarean section under general anesthesia.

No significant differences were observed in etomidate, remifentanil, and rocuronium bromide concentrations between term and preterm infants; twins and singletons are the main findings of this study. Etomidate has been used in cesarean sections under general anesthesia for many years. A previous study (Gregory and Davidson, 1991) demonstrated that the umbilical/maternal vein ratio of etomidate was 0.5 (SD 0.18), with no relation to time in the range encountered, and that the uterine artery/uterine vein ratio of etomidate was 0.86 (SD 0.33), suggesting that etomidate uptake into the fetus is effectively complete. Another study (Esener et al., 1992) in 40 patients undergoing cesarean deliveries found that the mean plasma etomidate concentration decreased rapidly (1,242.0 ng/mL at 5 min to undetectable 2 h after injection) and that the umbilical:maternal vein ratio was 1:24. And etomidate levels in colostrum were 79.3 ng/mL after 30 min, which reduced to undetectable after 4 h, indicating that a single injection of etomidate is safe for parturients and lactation.

Remifentanil is a μ-opioid receptor agonist with weak affinity to δ- and κ-receptors. Blood-brain balance is rapidly reached in the human body in approximately 1 min and the drug is rapidly hydrolyzed in tissue and blood, resulting in a fast onset and short maintenance time, which is obviously different from other fentanyl analogues. Remifentanil is mainly metabolized by non-specific esterase hydrolysis in plasma and tissues. Approximately 95% of remifentanil is metabolized and excreted in urine, with the main metabolite activity being only 1/4,600. There is no change in metabolic rate and no accumulation in the body after long-term infusion or repeated injection, and the incidence of delayed respiratory depression is very low (Kan et al., 1998; Sridharan and Sivaramakrishnan, 2019). A previous study (Welzing et al., 2011) demonstrated that very preterm infants exhibited a high nonspecific esterase activity in umbilical cord blood compared with term infants, which contributed to the rapid drug metabolization in preterm infants. This rapid metabolic feature also explains the results in our study, wherein the plasma concentrations of remifentanil and etomidate are not different between the groups. As for the positive correlation found between remifentanil plasma concentrations and neonatal weight in UA blood, it is inferred that preterm infants exhibit a high nonspecific esterase activity in umbilical cord blood compared with term infants, affecting the metabolism and distribution of remifentanil in newborns.

Rocuronium provides the shortest onset of action of non-depolarizing blocking agents, with few adverse reactions. It is highly water-soluble and difficult to pass through the placental barrier. As early as 1994, it was used for general anesthesia in cesarean section (Abouleish et al., 1994). Due to its characteristic large molecular weight, most studies related to rocuronium (Abu-Halaweh et al., 2007; Blaha et al., 2020) focused on the onset time and muscle relaxant effects, while the exact placental transfer rate was absent. Our results show that the placental transfer rate of rocuronium in combination with etomidate and remifentanil was 16% in single term neonates.

Apgar scores are commonly used as surrogate measures to predict subsequent adverse neonatal outcomes (Cnattingius et al., 2020). Due to the grouping conditions, the Apgar scores between groups were different for neonates, with those from twin premature delivery being inferior in most indicators (e.g., weight, the reflex and color scores of 1 min and 5 min Apgar scores, the proportion of using bag-mask ventilation and tracheal intubation, and umbilical artery blood pH). In addition, D-dimer was different (higher in twins) between groups due to the grouping conditions, which is consistent with the changing model results of D-dimer in twins (Bar et al., 2000; Morikawa et al., 2011).

This study is the first to describe the concentrations and placental transfer rates of intravenous drugs in twins and premature infants born by cesarean section under general anesthesia. However, the study has several limitations. Firstly, the sample size of the study is small, resulting in a large data standard deviation and some differences (such as the positive correlation between remifentanil concentration in UA and neonatal weight) that cannot be clearly explained. It is necessary to collect a larger sample size to clarify this confusion. Secondly, cord blood concentrations were determined only at birth, which does not reflect dynamic changes in the features of neonates after birth. In addition, monochorionic and dichorionic diamniotic twins were pooled for analysis, since obtaining a sufficient number of cases for twins under general anesthesia is challenging.

In conclusion, preterm or twin deliveries do not affect the neonatal concentrations of etomidate, remifentanil, and rocuronium bromide used in general anesthesia for cesarean sections.

## Data Availability

The original contributions presented in the study are included in the article/Supplementary Material, further inquiries can be directed to the corresponding author.
