# Supplementary material for: Anesthetic drug concentrations and placental transfer rate in fetus between term and preterm infants, twins, and singletons
Source: Front Pharmacol. 2023 Sep 1;14:1213734. doi: 10.3389/fphar.2023.1213734 (PMC10502316; doi:10.3389/fphar.2023.1213734)
Supplement: Supplementary file 1 [file Table1.docx]

**Supplementary table 1.**

**Hemodynamic indicators of parturients at different time points**

| Parameters | Group I  (n=38)  Full-term singleton | Group II  (n=10 )  Premature singleton | Group III  (n=5 )  Term twins | Group IV  (n=7)  Premature twins | P Value |
| --- | --- | --- | --- | --- | --- |
| Preoperative HR(bpm) | 88±13 | 97±19 | 91±8 | 87±18 | 0.365 |
| Preoperative SBP(mmHg) | 129±16 | 139±23 | 129±13 | 142±18 | 0.182 |
| Preoperative DBP(mmHg) | 83±12 | 79±19 | 82±11 | 91±10 | 0.280 |
| Preoperative SpO_2_ | 98±0.90 | 98±0.94 | 98±0.45 | 98±0.95 | 0.831 |
| HR at skin incision(bpm) | 96±19 | 96±18 | 96±6 | 102±16 | 0.895 |
| SBP at skin incision(mmHg) | 118±19 | 128±23 | 122±5 | 122±18 | 0.501 |
| DBP at skin incision(mmHg) | 74±14 | 77±17 | 75±8 | 79±11 | 0.842 |
| SpO_2_ at skin incision | 99.24±0.75 | 99.60±0.70 | 99.80±0.45 | 99.14±0.69 | 0.211 |
| HR at endotracheal intubation(bpm) | 94±15 | 99±20 | 96±4 | 97±12 | 0.787 |
| SBP at endotracheal intubation(mmHg) | 117±17 | 125±19 | 120±9 | 119±15 | 0.668 |
| DBP at endotracheal intubation(mmHg) | 72±13 | 65±23 | 74±7 | 74±7 | 0.536 |
| SpO_2_ at endotracheal intubation | 99.24±0.94 | 99.40±0.84 | 99.40±0.89 | 99.14±0.69 | 0.918 |
| HR at delivery(bpm) | 85±15 | 87±12 | 93±13 | 92±13 | 0.470 |
| SBP at delivery(mmHg) | 111±14 | 118±19 | 110±6 | 112±13 | 0.595 |
| DBP at delivery(mmHg) | 65±10 | 64±15 | 63±11 | 69±11 | 0.778 |
| SpO_2_ at delivery | 96.92±14.51 | 99.50±0.85 | 99.40±0.89 | 99±0.82 | 0.900 |
| HR at skin suture(bpm) | 78±12 | 82±10 | 84±10 | 87±14 | 0.228 |
| SBP at skin suture(mmHg) | 114±10 | 118±11 | 119±8 | 120±6 | 0.234 |
| DBP at skin suture(mmHg) | 68±10 | 68±10 | 71±7 | 75±11 | 0.319 |
| SpO_2_ at skin suture | 99.32±0.87 | 99.20±1.23 | 99.40±0.89 | 99.14±0.69 | 0.946 |

HR: heart rate, SBP: systolic blood pressure, DBP: diastolic blood pressure, SpO_2_: pulse oxygen saturation
