# Supplementary material for: Anesthetic drug concentrations and placental transfer rate in fetus between term and preterm infants, twins, and singletons
Source: Front Pharmacol. 2023 Sep 1;14:1213734. doi: 10.3389/fphar.2023.1213734 (PMC10502316; doi:10.3389/fphar.2023.1213734)
Supplement: Supplementary file 2 [file Table2.docx]

**Supplementary Table2.**

**Blood concentration of etomidate, remifentanil, rocuronium bromide between the classifications of twins**

| Parameters | | Monochorionic diamniotic  (MA n=2,  UV,UA, n=4) | Dichorionic diamniotic  (MA n=10,  UV,UA, n=20) | P value |
| --- | --- | --- | --- | --- |
| Etomidate | MA, ng/ml | 365.86±151.79 | 582.69±154.03 | 0.099 |
|  | UV, ng/ml | 229.59±59.15 | 360.94±125.83 | 0.066 |
|  | UA, ng/ml | 155.70±26.65 | 185.71±66.78 | 0.393 |
| Remifentanil | MA, ng/ml | 3.87±1.00 | 3.52±1.10 | 0.689 |
|  | UV, ng/ml | 1.63±0.43 | 1.58±0.71 | 0.896 |
|  | UA, ng/ml | 0.33±0.49 | 0.74±0.76 | 0.322 |
| Rocuronium bromide | MA, μg/ml | 4.76±2.09 | 6.78±3.10 | 0.406 |
|  | UV, μg/ml | 0.78±0.48 | 0.80±0.60 | 0.952 |
|  | UA, μg/ml | 0.43±0.28 | 0.41±0.27 | 0.896 |

MA: maternal arterial, UA: umbilical arterial, UV: umbilical venous
